# Developing an e-Prehabilitation System of Care for Young Adults Diagnosed With Cancer: User-Centered Design Study

**DOI:** 10.2196/41441

**Published:** 2023-03-30

**Authors:** Lisa McCann, Christopher Hewitt, Kathryn A McMillan

**Affiliations:** 1 Digital Health and Wellness Group University of Strathclyde Glasgow United Kingdom; 2 Clinical Health Psychology Astley Ainslie Hospital Edinburgh United Kingdom; 3 Project Management Office NHS Shetland Headquarters Lerwick United Kingdom

**Keywords:** digital health, human factors, user-centered, prehabilitation, young adults, cancer

## Abstract

**Background:**

A diagnosis of cancer in adolescence or young adulthood can pose many different and unique challenges for individuals, as well as their families and friends. Drawing on the concept of prehabilitation, the provision of high-quality, accessible, timely, reliable, and appropriate information, care, and support for young adults with cancer and their families is critical to ensure that they feel equipped and empowered to make informed decisions relating to their treatment and care. Increasingly, digital health interventions offer opportunities to augment current health care information and support provision. Co-designing these digital health interventions can help to ensure that they are meaningful and relevant to the patient cohort, thereby maximizing their accessibility and acceptability.

**Objective:**

This study had 4 primary interlinked objectives: understand the support needs of young adults with cancer at the time of diagnosis, understand the potential role of a digital health solution to assist in the delivery of prehabilitation for young adults with cancer, identify appropriate technologies and technological platforms for a digital prehabilitation system of care, and develop a prototype for a digital prehabilitation system of care.

**Methods:**

This was a qualitative study using interviews and surveys. Young adults aged 16 to 26 years diagnosed with cancer within the last 3 years were invited to participate in individual user-requirement interviews or surveys. Health care professionals specializing in the treatment and care of young adults with cancer and digital health professionals working in the industry were also interviewed or completed a survey. Consensus feedback interviews were conducted with 3 young adults and 2 health care professionals after the development of the first generation of the prototype app.

**Results:**

In total, 7 individual interviews and 8 surveys were completed with young adults with a range of cancer diagnoses. Moreover, 6 individual interviews and 9 surveys were completed with health care professionals, and 3 digital health professionals participated in one-on-one interviews. A prototype app with the working name of Cancer Helpmate was developed based on these collective participant data. Overall, feedback from participants across the data collection activities suggests that the concept for the app was positive during these developmental stages. Further insightful ideas for the app’s future development were also identified.

**Conclusions:**

Young adults with cancer and health care professionals are responsive to the need for more digitally driven services to be developed. Further development of an app such as Cancer Helpmate, which incorporates key features and functionalities directly informed by users, could help to augment the support provided to young adults with cancer.

## Introduction

### Prehabilitation

More than 1.3 million adolescents and young adults (YAs)—individuals aged 15 to 39 years—were newly diagnosed with cancer globally in 2019 [1]. In the United Kingdom, where YAs with cancer are referred to as “teenagers and young adults” and are typically aged 15 to 24 years, <1% of new cancer cases are diagnosed in this population, making it a relatively rare illness [2]. However, it is well established that the cancer burden as well as experiences of treatments and their associated side effects can present different challenges when compared with those involving an older population [1] because of physical, psychosocial, educational, and financial challenges associated with a cancer diagnosis and treatment at this particular developmental life stage [3-5]. A diagnosis of cancer at any point will always cause some biographical disruption to an individual, but during adolescence and young adulthood in particular, there can be substantial disruption to developmental milestones, education, career, relationships, self-esteem, body image, and identity [6]. By definition, life experience will be shorter in YAs, and therefore opportunities to develop and rehearse robust coping strategies will generally be more limited [4,7].

Research has highlighted the importance of providing specialized information, care, and support to and for YAs and their families at the time of cancer diagnosis [8,9]. Attention is rightly focused on the psychological well-being and resilience of YAs, with some evidence suggesting that developing resilience in the initial stages of a cancer diagnosis and treatment may aid longer-term coping [3]. In this regard, there has been a move toward delivering interventions—physical, diet, and psychosocial—in the interim period between diagnosis and treatment commencement. This concept is now commonly described as prehabilitation [10].

Historically, prehabilitation efforts focused on maximizing a patient’s physical fitness (eg, for surgery), with the aim of having a positive impact on survival, coping skills, and patient-reported outcomes during and after treatment [11-15]. However, the concept of prehabilitation in the context of cancer care has gathered momentum and is now recognized as an increasingly important area of cancer supportive care provision. In the United Kingdom, in November 2020 [16], MacMillan Cancer Support published guidance advocating the use of prehabilitation (both for physical and psychological needs) in the management of, and support for, people living with cancer. The report specifies a series of prehabilitation principles, with 3 key benefits from the inclusion of prehabilitation in cancer care provision identified. These were as follows: (1) personal empowerment and a sense of control for the patient, (2) physical and psychological resilience and improved quality of recovery from treatment, and (3) a positive impact on long-term health through positive changes in behavior [16]. This guidance advocates for prehabilitation to coexist within the rehabilitation pathway from either the point of diagnosis or even before the diagnosis in some cases so that people diagnosed with cancer can be best prepared, physically and mentally, for treatments and later stages of the cancer pathway [16]. However, the development of tailored prehabilitation interventions and associated supportive care services for patient populations such as YAs with cancer is in its infancy.

### User-Centered Design Processes for Developing Prototype Digital Health Interventions

User-centered design processes are those defined by collaborative, cooperative, and cocreation methods; thus, they lend themselves well to the development of new interventions that are responsive to and meet the needs of target populations. Therefore, a user-centered design backdrop, set within the rapidly evolving digital health agenda, enables researchers to explore opportunities to tackle current and future health care challenges via technology-based interventions. The uptake of digital health interventions, including those that use technologies such as websites, mobile apps, and wearables, has accelerated in recent years, particularly in the period from 2020 to 2022 during the global COVID-19 pandemic [17-19]. Assumptions of data literacy among YAs and their access to and use of technologies in health care contexts [20] present opportunities to better identify meaningful technology-based interventions in health care provision for YAs with cancer [21,22].

User-centered design methodologies are invaluable in identifying and designing acceptable and appropriate interventions for target populations [23]. They afford some flexibility, are typically iterative in nature, and allow for critical contextual insights to inform and direct the design, development, and evaluation of digital solutions and interventions through their 3 typically classified categories of inspection, testing, and inquiry [24]. Thus, applying a user-centered design approach to the development of a mobile phone app to support prehabilitation in YA cancer care has the potential to help improve the experiences of this patient cohort.

**Figure 1:**
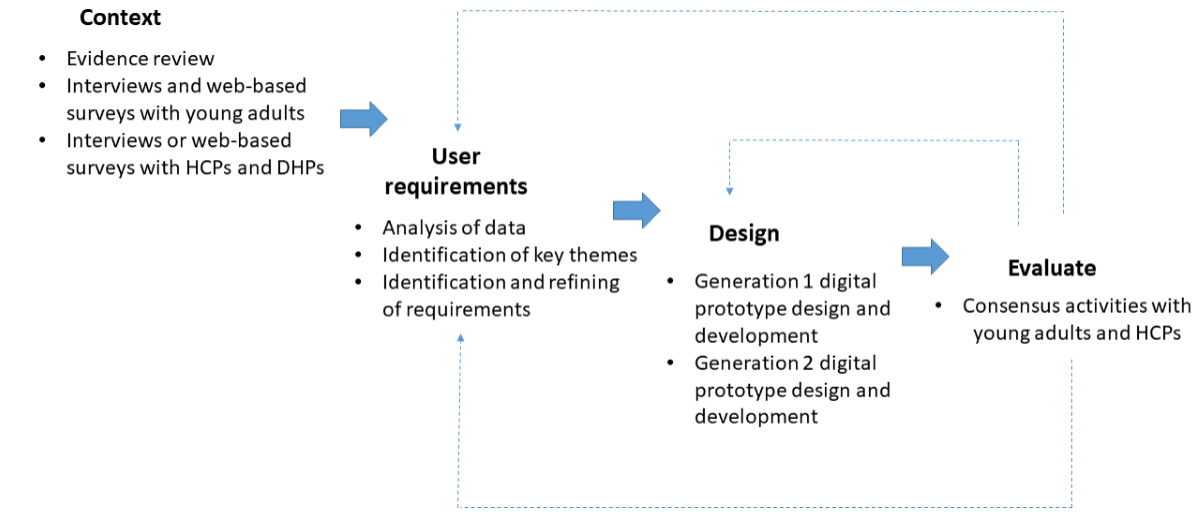
The user-centered design process. DHP: digital health professional; HCP: health care professional.

This paper presents an overview of the user-centered design process in the development of a prototype mobile phone app (working name: *Cancer Helpmate*) to support prehabilitation experiences of YAs diagnosed with cancer (Figure 1). This paper reports on the contextual understanding of YAs’ diagnosis experiences and pathways of care, and the initial user-centered development rounds of a digital solution focused on addressing these needs.

## Methods

### Study Design

This was a qualitative user-centered design project. The protocol paper for this project has been published previously [25]. To ensure that the prototype product design and purpose were meaningful and informed directly by users’ requirements and experiential insights, the research team recruited key stakeholders, including YAs with cancer, health care professionals (HCPs), and digital health professionals (DHPs), to the study.

### Ethics Approval

The study received full ethics approval from the Yorkshire and the Humber–Bradford Leeds Research Ethics Committee in the United Kingdom (17/YH/0352) and was endorsed by the University of Strathclyde Ethics Committee shortly thereafter. Local research and development management approval (GN17ON664) was received for the study, as was approval from the participating cancer hospital before study commencement. No financial or incentive payments were made for participation.

### Recruitment

#### YA Participants

Using purposive sampling, YAs aged 16 to 26 years diagnosed with cancer up to 3 years but no less than 4 weeks before participation at the time of recruitment were invited to participate in this study by the YA cancer team at the partner cancer hospital. HCPs from the cancer team identified and approached potential YA participants either in person or via email by reviewing clinic lists, caseloads, and databases. Potential participants were introduced to the study by the cancer team through age-appropriate study information, provided in written and video formats on a study-dedicated website. YAs interested in participating were asked to complete a consent-to-approach form or contact the research team directly with their contact details. The research team then contacted the YAs to discuss participation and confirm eligibility to participate.

A range of self-referral recruitment methods were also used to recruit YAs to the study, including placing advertisement posters and postcards around university buildings and the hospital’s clinics, recruitment-orientated posts on a dedicated project Twitter account, development and use of a dedicated project website, and contacting YA-specific support groups. If YAs were interested in participating in the study after learning of it via one of these self-referral channels, they contacted the research team directly via the study email address or completed a screening survey on the study website to confirm their eligibility. The research team followed up with the individual thereafter to review and confirm participation.

Recruitment was more challenging than anticipated, even with the simultaneous activation of the aforementioned recruitment strategies. In response, an additional recruitment strategy was implemented after an ethics protocol amendment. In addition to the existing strategies, a member of the research team established a presence at the YA clinics at the cancer hospital. This visibility enabled potential participants to have a face-to-face introductory dialogue with the researcher about the study immediately after initial introduction from the HCP, and this strategy helped to enhance recruitment.

#### HCP and DHP Participants

Researchers purposively identified and directly approached HCPs with experience of working with YAs with cancer. DHPs—individuals with experience of developing and deploying digital health solutions and interventions within National Health Service, industry, and academic contexts—were also approached directly by the research team to offer their perspectives as domain experts.

Inclusion criteria for each of the participant groups are summarized in Textbox 1.

**Participant inclusion criteria.**

- Teenagers and young adults (YAs)
  - Aged 16 to 26 years
  - Diagnosed with cancer up to 3 years but no less than 4 weeks before participation
  - Receiving or received services by National Health Services Scotland or [hospital name]
  - Sufficiently proficient in English to be able to participate in data collection activities
- Health care professionals
  - Member of the teenagers and YAs cancer team or multidisciplinary team involved in the provision of care to YA with cancer
  - Have experience working with YA who have or have had a diagnosis of cancer
  - Sufficiently proficient in English to be able to participate in data collection activities
  - Able to provide informed consent
- Digital health professionals
  - Professionals with experience of working in the digital health space within National Health Service, industry, and academic contexts
  - Sufficiently proficient in English to be able to participate in data collection activities
  - Able to provide informed consent

### Data Collection Activities

This study used the concept of user-centered design—an iterative approach to product design and development that evolves through cycles of contextual understanding, requirements capture, design and development, and evaluation—and qualitative data collection activities to address the study objectives. This is illustrated in Figure 1, which has drawn inspiration from the user-centered design framework as advocated by the Interaction Design Foundation [26].

#### Stream 1: Interviews or Web-Based Surveys With YAs

Individual interviews or web-based surveys were completed by YAs who were currently or previously diagnosed with cancer to develop an understanding of the issues they experienced before and upon diagnosis. The interviews lasted between 46 and 60 minutes, and survey completion time ranged from 4 to 45 minutes. The aim was to recruit up to 20 YAs who met the inclusion criteria to participate in the first stage of data collection via these 2 data collection strategies.

Within the interviews and surveys, YAs were asked about their experiences regarding the following aspects:

- The experiential impact of cancer diagnosis within the context of the following themes:
  Practical
  Family
  Emotional
  Social
  Physical
- Current cancer information provision
- Suggestions for the role of technology to support YAs newly diagnosed with cancer

#### Stream 2: Interviews or Web-Based Surveys With HCPs and DHPs

In stream 2, which ran concurrently with stream 1, we provided HCPs and DHPs with specific participant information sheets and invited them to participate in one-on-one interviews or complete a web-based survey to explore their preferences for the content and delivery of the system. Upon consent, the interviews lasted for approximately 60 minutes. The aim was to recruit up to 21 individuals, with 2 to 3 representatives from each professional group, including nursing, oncology and hematology, psychology, physiotherapy, occupational therapy, social work, and youth support, as well as professionals working with digital health solutions and interventions from health care, industry, and academic settings.

Within these interviews and surveys, HCPs and DHPs were asked about the following topics:

- Their experiences of information provision before initiation of YA cancer treatment
- Preferences and requirements for the e-prehabilitation resources and materials
- Preferences for the technology system
- The role of an e-prehabilitation system in assisting in the prehabilitation care offered

#### Stream 3: Prototype Design and Development

Data gathered in streams 1 and 2 informed the user and system requirements for the first generation of the prototype. A medium- to high-fidelity prototype of a web-based mobile app that could be viewed and evaluated by users was developed at the end of the first development cycle (ie, generation 1 of the product).

#### Stream 4: Consensus Activities

In stream 4, we sought feedback from participants on the generation 1 medium- to high-fidelity prototype developed in stream 3 so that we could review our interpretation and development of the prototype derived from our user requirements, experience, and insights.

We did this by creating environments—paper and digital—for participants (YAs and HCPs) to access and comment on the medium- to high-fidelity prototype. Where this was not possible, we distributed an electronic version of the prototype by email along with a link to a web-based survey and asked for comments and feedback. Participation in stream 4 was optional for both YA and HCP participants.

### Data Analysis

Interviews were audio recorded and transcribed verbatim using an external transcriber. Transcripts were merged with field notes and outputs of brainstorming activities. During the analysis, 2 researchers (LM and KM) drew upon the research objectives and identified and developed themed categories to guide the data analysis. NVivo (version 12; QSR International), a qualitative analysis software package, was used to support the data analysis activities.

Data were analyzed using a thematic analysis approach because this is useful for answering questions about the salient issues for a particular group of respondents or for identifying typical responses [27]. For reliability and validity purposes, 2 researchers (LM and KM) coded a subsample of transcripts and field notes separately and then cross-checked them together. The remaining transcripts and field notes were coded by researcher KM.

## Results

### Streams 1 and 2: Participant Demographics

#### YA Participants

In stream 1, a total of 7 YAs participated in interviews with a member of the research team. The mean age of the YAs at the time of participation was 21.7 (SD 3.2) years, and their mean age at the time of cancer diagnosis was 20.5 (SD 2.8) years. Most of the YAs (5/7, 71%) received their treatment in the specialist YA cancer ward at the partner cancer hospital in Scotland, and all participants received chemotherapy as part of their treatment, with surgery (4/7, 57%) and radiotherapy (1/7, 14%) also being received. All participants received a pack of information materials provided by the clinical nurse specialist at the time of diagnosis. Other information resources used by the YAs at this time included websites (6/7, 86%), social media (3/7, 43%), and personal blogs (1/7, 14%). Participant demographics from the sample who participated in the interviews are outlined in Table 1.

The web-based survey contained a set of initial screening questions to facilitate immediate completion by those YAs who met the inclusion criteria. In total, 17 YAs started the web-based survey, but the initial screening questions identified the following concerns: 1 (6%) was too old, 2 (12%) did not fit the diagnosis criteria, and 1 (6%) did not complete the screening survey. Of the remaining 13 YAs who were eligible to complete the remainder of the main web-based survey, only 8 (62%) actually continued beyond the screening survey. Of these 8 YAs, 6 (75%) completed the web-based survey, whereas 2 (25%) only partially completed it. Specific demographic data beyond the eligibility screening criteria (aged 16-26 years, diagnosed with cancer 1-36 months ago, and treatment commenced 1-36 months ago) were not collected.

Questions asked within the web-based survey were open-text questions, and the free-text qualitative data from the survey participants (n=8) were integrated with the interview data in stream 3.

**Table 1:** Young adult interview demographic information (N=6^a^).

Variable		Values		
Current age (years), mean (SD)		21.7 (3.2)		
Age at diagnosis (years), mean (SD)		20.5 (2.8)		
**Sex, n (%)**				
	Male	3 (50)		
	Female	3 (50)		
**Education level, n (%)**				
	Higher or A level or SVQ3^b^	2 (33)		
	Advanced higher or certificate of higher education	2 (33)		
	Honors degree	1 (17)		
	Master’s degree	1 (17)		
**Employment status, n (%)**				
	Full time	3 (50)		
	Part time	2 (33)		
	Seeking work or student	1 (17)		
**Relationship status, n (%)**				
	Single	4 (67)		
	Living with partner	2 (33)		
**Cancer type, n (%)**				
	Sarcoma	1 (17)		
	Testicular cancer	1 (17)		
	Lymphoma^c^	4 (67)		
**Period elapsed since diagnosis (months), n (%)**				
	3 to 5	1 (17)		
	6 to 9	1 (17)		
	12 to 24	4 (67)		
**Treatment received, n (%)**				
	Surgery	4 (67)		
	Radiotherapy	1 (17)		
	Chemotherapy	6 (100)		
**Receiving cancer treatment currently, n (%)**				
	Yes	1 (17)		
	No	5 (83)		
**Where participants received most of their treatment, n (%)**				
	Regional cancer center			5 (83)
	Teenage Cancer Trust ward at regional cancer center			4 (67)
	Local hospital			1 (17)
**Information resources used at diagnosis, n (%)**				
	Print		6 (100)	
	Pack of materials provided by clinical nurse specialist		6 (100)	
	Websites		6 (100)	
	Social media		3 (50)	
	Blogs		1 (17)	

^a^Overall, 7 young adults were recruited, but 1 (14%) did not complete the demographic form.

^b^SVQ3: Scottish Vocational Qualification level 3.

^c^n=2: Hodgkin lymphoma, n=1: Burkitt lymphoma, and n=1: non-Hodgkin lymphoma.

#### HCP and DHP Participants

In stream 2, of the 15 HCP participants, 6 (40%) participated in interviews, and 9 (60%) completed a survey. In addition, 3 DHPs participated in interviews. In terms of the HCP interview participants, psychology, oncology, and allied health professions disciplines were represented, and the mean experience in their current role was 8.7 (SD 5) years, whereas the mean experience working with YAs with cancer was 5.1 (SD 5.7) years. Half (3/6, 50%) of the HCP interview participants had received specialist training for working with YAs with cancer. Further participant demographics are outlined in Table 2. The free-text qualitative data from the 9 surveys completed by HCPs were integrated with the interview data in stream 3.

**Table 2:** Health care professional (HCP) and digital health professional (DHP) interview demographic information (N=8^a^).

Variable		Values
**Age (years), n (%)**		
	25 to 34	1 (13)
	35 to 44	5 (63)
	45 to 54	2 (25)
**Profession, n (%)**		
	HCP	6 (75)
	DHP	2^a^ (25)
**Sex, n (%)**		
	Male	4 (50)
	Female	4 (50)
**Education level, n (%)**		
	PhD	4 (50)
	Medical degree	1 (13)
	Master’s degree	1 (13)
	Honors degree	2 (25)
HCP experience in current role (years), mean (SD)		8.7 (5)
HCP experience working with YAs^b^ with cancer (years), mean (SD)		5.1 (5.7)
DHP experience in current role (years), mean (SD)		2.8 (1.3)
DHP experience working with YAs with cancer (years), mean (SD)		5.5 (1.5)
**Specialist training to work with** **YAs** **with cancer, n (%)**		
	Yes	3 (38)
	No	5 (63)

^a^Overall, 3 digital health professionals were recruited, but 1 (33%) did not complete the demographic form.

^b^YA: young adult.

### Stream 3: Synthesis of Qualitative Data

The identified main and supporting subthemes identified deductively during data analyses of the collective interviews and surveys are summarized in Textbox 2. These themes and subthemes are further elaborated on in the following sections; evidence is provided with embedded key quotations for illustrative purposes.

**Main and supporting subthemes from interviews.**

- Needs of young adults at diagnosis
  - Diagnosis experience
  - Life disruption
  - Physical and psychological impact of cancer diagnosis
  - Information provision and delivery
- The role of technology to support prehabilitation in young adults with cancer
  - Understanding prehabilitation for young adults with cancer
  - Barriers and facilitators for technology use
  - Design and delivery of an e-prehabilitation system of care for young adults with cancer

#### Needs of YAs at Diagnosis

##### Diagnosis Experience

YA narratives illustrated that the period from initial symptomatic presentation to a confirmed cancer diagnosis was long and often lasted many months. YAs described this as a confusing and worrying time. There were some observed similarities among participants in this regard (ie, multiple presentations to a general practitioner and repeated referrals for tests with different specialists before receiving a definitive cancer diagnosis). From there, active treatment commenced at pace:

[T]he build-up to getting diagnosed was extremely long. I’d been ill since about before Christmas, and I kept going to the doctor’s and getting blood tests, and they were presenting me as anemic, and then I was having iron tablets, iron supplements, and then I went to get my blood tests again and it didn’t improve, and because of the timescale of me travelling...my doctor like referred me to hematology really quick, and then that’s what happened, but for months, I was actually meant to get my tonsils removed, they put it down to that; I was meant to get a tonsillectomy this month...but it took months to diagnose it, but once it was diagnosed, it was extremely fast.

YA001, 17 years old, female

So it was quite a shock, because I never really thought, well, I went in with a sore stomach and came out with cancer! It’s a bit of a strange scenario. So...yeah, it was a little bit daunting.

YA002, 20 years old, male

##### Life Disruption

YAs’ narratives revealed the different aspects of their lives disrupted by their cancer diagnosis, including relationships with friends and family, school and university, work, finances and planned holidays, and life experiences. YAs spoke about some of the challenges of maintaining friendships when they were not feeling well enough to engage in social activities. Participants spoke candidly about the impact of their diagnosis on their social networks and expectations of friends:

I think my relationship with my friends, at the beginning, it was different to the way it was throughout treatment, at the beginning: I wanted my friends around all the time, and then throughout treatment, I was just kind of, I was too tired to really socialize, and then after treatment, I think they were all under the impression as well that I would just like go back to the normal way of things and be going out at the weekends and things like that, but it was still like a hard transition to feeling normal again.

YA007, female

HCPs also acknowledged the range of impacts and life disruptions a diagnosis of cancer can have on a YA’s life; many identified issues similar to the following reflections:

Psychological challenges e.g. shattered assumptions about the future, mood/anxiety issues, body image concerns, fears of various treatment procedures, fears of dying. Relationship issues e.g. difficulties in intimate relationships, worries about how parents are coping, worries about how to tell friends re their diagnosis. Practical concerns e.g. interference with work/study/life plans, limiting ability to travel/see the world, financial worries. Worries about various symptoms and how they will cope e.g. nausea, pain, fatigue. Spiritual concerns where relevant.

HCP survey participant 05

##### Physical and Psychosocial Impact of Cancer Diagnosis

Given the emphasis on physical and mental health during prehabilitation, the interviews explored the physical and psychological impacts of a diagnosis of cancer on YAs. Most of the participants discussed the physical symptomatic impacts of a cancer diagnosis and treatments, including fatigue, nausea, diarrhea, pain, and weight changes. Hair loss, a physical manifestation of some cancer treatments, also had considerable psychological impacts because the anticipation of losing their hair caused considerable anxiety for YAs:

Every time I’d go to sleep, I’d be anxious to wake up in the morning to see if it [hair] had fallen out or not. Every time I went for a bath, I’d dread it, because it would all come out.

YA001, 17 years, female

YAs spoke with honesty about the initial shock and disbelief of being diagnosed with cancer. For some of the YAs, understanding their diagnosis information was compounded by the overwhelming amount of cancer-related information provided by their clinical team. Participants noted how this information could vary from focusing on expected treatment-related side effects to potential decisive life-course decisions such as fertility choices:

It’s more just like it’s such a short amount of time to understand anything. Like understanding the fact alone that you have cancer, because it’s such a big word when you’re not really involved with it...it’s such a big, scary word—so it’s just getting to terms with the fact that that’s what’s wrong with you, and then trying to understand how serious or like how treatable it is so quickly, as well, that’s quite big.

YA003, 25 years old, female

And I think it’s really important to think not just about physical side effects in their own right, but the psychological impact of a physical side effect, so how does it feel emotionally to feel so fatigued all the time? How does it affect your body image if you lose your hair, for example, or if your weight and muscle mass changes? It’s thinking kind of about the emotional impact of the physical symptoms: I think that’s kind of one step that’s sort of missed out sometimes, so there’s all of those things.

HCP002

##### Information Provision and Delivery

YAs and HCPs perceived current information provision at diagnosis to be very good; an information pack of written materials was provided as standard to YAs at the time of diagnosis from the participating hospital site. However, YAs repeatedly commented that the nature and presentation format of this information *“*can be very overwhelming, it is a lot of new information to take on-board in such a short space of time” (YA survey participant 01). As a result, engagement with the aforementioned materials was limited to the window between diagnosis and treatment commencement:

It was just kind of like information just thrown at you, and a lot of kind of leaflets, there were other books, but I couldn’t get my head round it, it wasn’t something you could take a read of.

YA005, 26 years old, male

At the beginning, I got this massive like pack of leaflets and pamphlets, and it was just too much literature that I didn’t read it all, just because it was so much: it was kind of overwhelming and I didn’t really know what I wanted to find out about.

YA007, female

They [information pack] were alright. Some of them, one of them was quite childish, one of the books. I always remember one being quite childish.

YA001, 17 years old, female

It was apparent that accessing accurate and reliable information drove some of the YAs to seek out their own information, predominantly from well-known cancer charity websites, social media, or blogs. However, there was a substantial gap in information provision–related experiences shared by peers:

Yeah, that was me going out and looking for it myself. The only thing that I was given was the leaflets and the websites, and the websites were great, but I would always look for more! I wanted to hear more and hear what other people were going through, and that’s when I started to hunt for the blogs and even these, not chat rooms, forums and things, like they were really helpful too: hearing how other people cope.

YA004, 19 years old, female

I feel that there is a lot of information out there, but sometimes not in one cohesive location in a format that people find accessible.

HCP survey participant 05

#### The Role of Technology to Support Prehabilitation in YAs With Cancer

##### Understanding Prehabilitation for YAs With Cancer

In the interviews, most YAs and HCPs talked about the realities of a very short period between cancer diagnosis and treatment commencement. Usually, prehabilitation focuses on a prolonged period before treatment commencement and on physical fitness and physical readiness for the surgery. However, in the context of this study, the focus was placed more on facilitating psychological readiness by providing appropriate information at the time of diagnosis and making this information available throughout treatment. Providing this support digitally was perceived to be a potential enabler of this care:

Years ago, when this idea was kind of in my mind, what triggered that was that I was aware of young people and their families kind of saying to me, “I wish I kind of knew then what I know now,” and I always think, that’s important, because that could really help people in the future. But, in reality, the window was going to be too small, because between being diagnosed and starting treatment, often there isn’t very much opportunity, and people are geographically spread. So, that’s when we were looking at an electronic format.

HCP001

##### Facilitators and Barriers for Technology Use

All YAs interviewed spoke about the role and presence of technologies such as mobile phones and the internet in their everyday lives, especially in relation to seeking information relative to their cancer experience, treatments, and side effects. Technology was perceived to have a positive role in health care, particularly in facilitating access to information and support:

I think the benefit to them [apps] is particularly if you’re encouraging somebody to do some sort of self-monitoring, I think most people have got their mobile phone on their person 24/7, so there’s definitely a benefit to that, versus if you give somebody like a diary, they’re not going to really have that about with them, so then you miss information.

HCP002

Yeah, I think it probably would help, because then you’ve got—especially if it’s a more kind of central place to get information, it’s more, like a lot of the sites that are there will link to other sites more, but then quite often you’ll find that you’ll be going back and forward between the same kind of sites, whereas if you’ve got somewhere central you can go that kind of gives you more specific information or, yeah, something like that.

YA002, 20 years, male

Accessibility was also identified as a facilitator for using technology to support YAs; providing the same amount of information in an app or website as in a written format was considered by YAs to be more accessible and less overwhelming:

Yeah. Something that’s like accessible. A big pile of papers is accessible, if you want to go through all the information, but not necessarily everybody does, and I know now, even for everything in my life, if I’m looking for information on something, I’m on my phone, like I’m looking for something that’s going to give me information on it straight away...So, it makes sense to just have another app or another website that just has, it just fulfils another need for people who have questions.

YA003, 25 years, female

YAs described using an app on their phone as easier than going through printed materials when they were feeling nauseous or fatigued because it requires less effort to *scroll* than sift through multiple papers. They also highlighted that having a single app where all the information was collated would reduce the overlap of information, which they often found was the case with leaflets and books from multiple sources. This would also provide the ability to filter the information so that the user is able to access the information most relevant to them and their situation, which can be done much more quickly electronically than with paper formats.

##### Design and Delivery of an e-Prehabilitation System of Care for YAs With Cancer

The preferred form of an e-system suggested by all participants was a mobile app that could be used on both iOS and Android devices. The participants identified system features and functionalities as well as design and delivery. A repeated theme was the need for the app to be personalized to the user in some way. Suggestions around personalization included the ability of the user to personalize how the system looked (colors, text size, and font) and the ability to personalize and tailor the information that was presented to them. Other design suggestions concerned the importance of the e-system being engaging through the use of bright colors, a catchy name, and a combination of media for the way information was presented (text, pictures, and video):

High quality, professionally designed, very functional app accessible on both IOS and Android. If only provided as website, there are already numerous alternatives to use. If the quality is not better than other existing resources online, TYAs [teenagers and young adults] will not use it. For most TYAs a gimmick is not required.

HCP003

I think kind of bright colors, to make it a bit more engaging, and maybe visuals, that would help, I think. I think a lot of text can be kind of overwhelming sometimes, so maybe videos of different things and images, I think that would help.

YA007, female

For the YAs, the most important and consistently identified functionality, alongside the provision of tailored information, was the inclusion of peer and professional experience stories. Some of the YAs spoke of self-seeking this information by accessing blogs and social media posts of other YAs. Others spoke about their desire for this sort of information to have been made available to them:

I don’t think you necessarily want someone to come and talk to you like this, but being able to read someone’s situation, I know I did that a lot...You kind of want to read that information, because you don’t always know how to process what’s happening, so yeah, other people’s experiences definitely help...I just think it’s more personal. Like a lot of the information that you get isn’t personal, it’s clinical.

YA003, 25 years old, female

Having someone to chat to, who has been through it before who can help provide reassurance.

YA survey participant 07

Reassurance. From professionals and people who are relatable, gone through it themselves, of similar age. One of the biggest thing that help me through my journey was meeting a girl of the same age with the same cancer but a year ahead of me. Seeing the other side helped me remain positive.

YA survey participant 01

Further design considerations for the app were identified during discussions with YAs about their own coping mechanisms; for example, a YA discussed some of the “self-hacks” she used to track her diagnosis and treatment pathway, including the use of lettering on light boxes and daily manual updates to the numbers on the light box. It was suggested that incorporating a digital timeline principle into the app may provide individuals with some personalized information about their forthcoming treatment pathway upon diagnosis:

Do you know what would also be really good? I have—I don’t know if you see it over there—it’s a wee light-up box, it says Hodgkin’s Fighter. It used to have how many days since I’ve been diagnosed, but now it’s 31 days until my last chemotherapy. So it’s counting it down...What would be really cool is if you put in an estimated date of the last chemotherapy, and they can see that number reduce daily. That would be really cool...it could be like a timeline, so it could be like, “Oh, I was admitted for tonsillitis this day, chemotherapy postponed a week,” and then it would update it for you and it would tell you how many days you had left to go. That’s motivating, because I can finally see a finish line.

YA001, 17 years old, female

### Stream 3: Prototype Design

**Figure 2:**
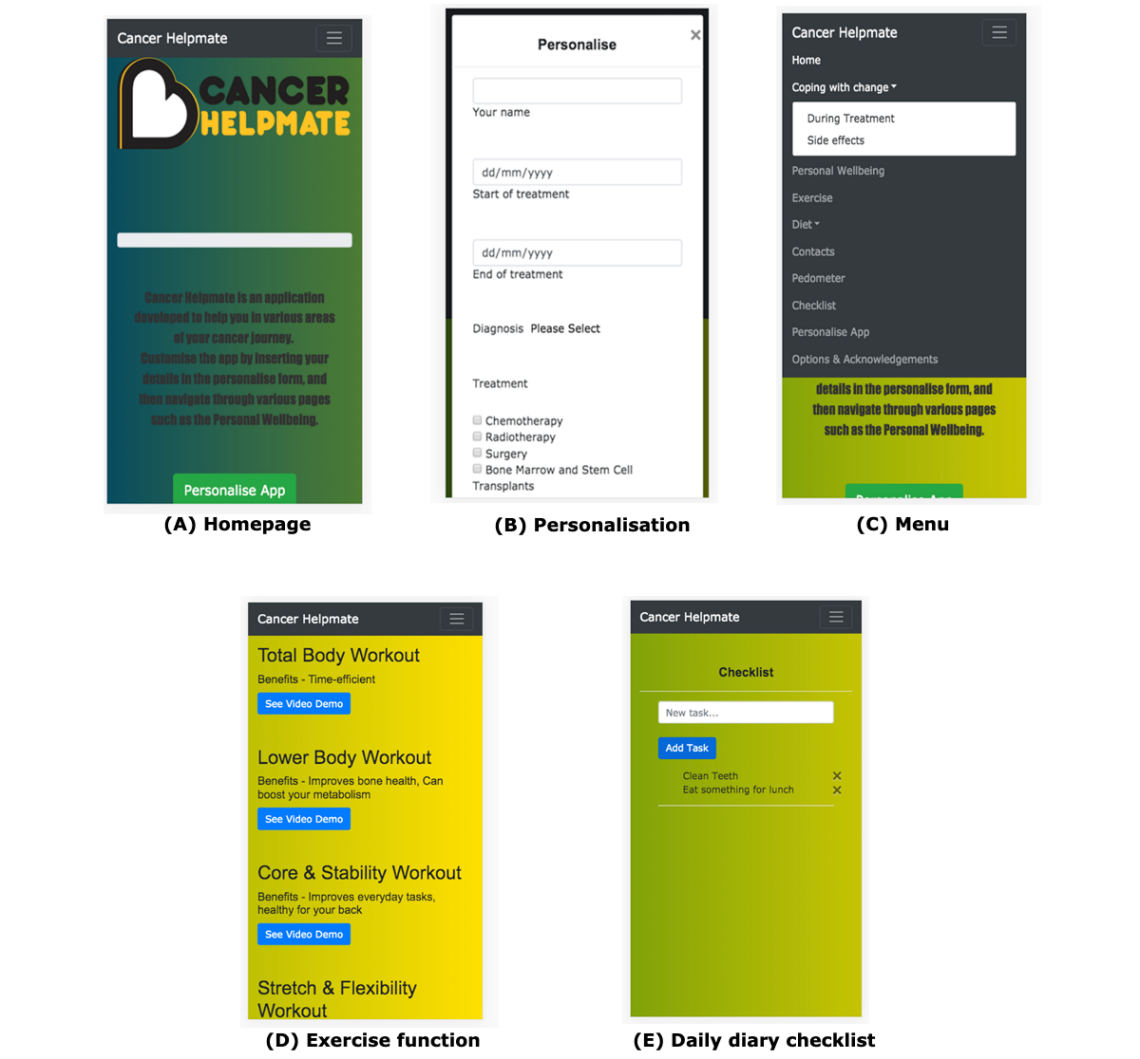
Cancer Helpmate app prototype (generation 1).

Analyzed data informed the requirements for the development of generation 1 of the medium- to high-fidelity prototype app. Table 3 summarizes these identified key user requirements for the prototype, the user-experience source, and the implementation outcome of these requirements in generation 1 of the prototype. Sample screenshots of generation 1 of the prototype are illustrated in Figures 2A to 2E. Cancer Helpmate was chosen as the working name for the product to reflect feedback from participants in the interviews and because they particularly requested that the name explicitly reference cancer as the primary purpose of the app.

**Table 3:** Prototype product requirements (generation 1).

Key user requirements	User-experience source	Implementation outcome (in generation 1 of Cancer Helpmate)
Cross-device product	Young adults	Mobile app developed, accessible via URL and a QR code, usable on mobile phones and tablet devices
Product can be personalized	Young adults and HCPs^a^	App interface can be personalized on registration and log-in by users by answering some brief questions on the app landing page about their specific diagnosis (Figure 2B)
Diagnosis information can be personalized on the product	Young adults	Personalizing app interface on registration and log-in means diagnostic-specific information will be presented to user, rather than generic cancer information
Treatment-related information can be personalized on the product	Young adults	Personalizing app interface on registration and log-in means information on relevant treatment-related symptoms and side effects a person may experience and how to go about seeking help is provided
Dietary information provided	Young adults	A diet function allows users to see a range of recommended healthy recipes and meals, with links to websites that will teach them how to make them at home; also has functionality for a user to log their own recipes and meals and store these in the app
Exercise information provided	Young adults	Exercise functionality includes text and links to video tutorials and demonstrations to recommended exercises for use during treatment and survivorship phases (Figure 2D)
Treatment countdown clock	Young adults	Countdown clock functionality incorporated: the user enters their expected treatment end date upon app registration, and functionality provides a clear-visual daily countdown visualization until the end of treatment
Simple pedometer function	Young adults	Simple pedometer function integrated; illustrates number of steps walked, but with drop-down menu to self-select realistic targets each day based on symptom experiences
Daily diary checklist	Young adults	Daily diary checklist functionality embedded: users can add their own self-directed tasks to a list and score them out once completed (Figure 2E)
Inclusion of reliable and trustworthy information	Young adults and HCPs	Inclusion of links to existing teenagers and young adults cancer charities and collated contact details for these organizations
Centralized information about cancer in 1 place	Young adults and HCPs	App includes menu options for information about cancer diagnoses, treatments, self-care advice, and cancer support organizations

^a^HCP: health care professional.

### Stream 4: Consensus Feedback Activities

The consensus feedback activities provided us with an opportunity to not only confirm the acceptability of the features and functionalities of generation 1 of the app but also identify more features and functionalities to include in generation 2 and its subsequent design and development cycle.

**Figure 3:**
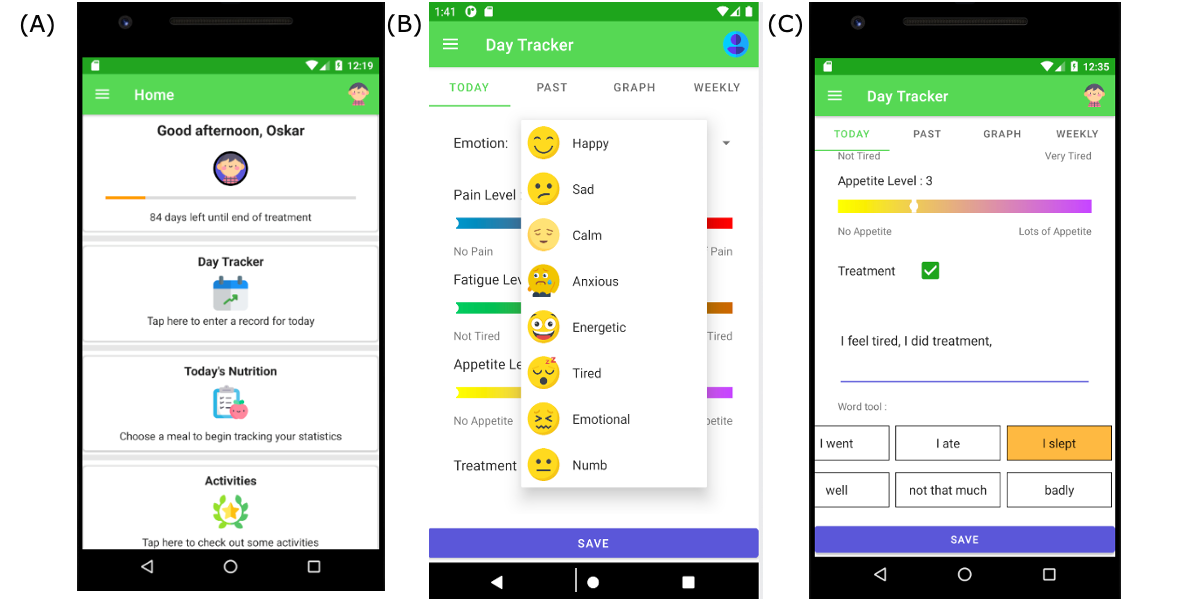
Cancer Helpmate app prototype (generation 2). (A) Home page. (B) Daily tracker and emotions assessment. (C) Daily tracker word wall.

The YAs (n=7) and HCPs (n=6) who participated in the study interviews were invited to provide feedback on the prototype. Of the 7 YAs, 3 (43%) participated in these consensus feedback activities (one-on-one interactions), where they reviewed the prototype with the researcher. Of the 6 HCPs, 2 (33%) participated together and provided their input collectively during their review of the prototype with the researcher. The current features, functionalities, and design of the app were reviewed, in turn, with participants asked in a *think-aloud* approach for the considered strengths and limitations of the current version of the app. During such directed conversations, participants were also asked to *think aloud* about what changes or additions would be beneficial to make to the next generation of the Cancer Helpmate app. The information was recorded by the researcher during each interaction and is summarized in Textbox 3; examples of this actioned feedback are presented in Figures 3A to 3C.

**Summarized feedback on generation 1 of Cancer Helpmate.**

- Suggested changes and additions for generation 2 of Cancer Helpmate
  - Include section on experience of peers and way to interact with peers
  - Better use of color throughout the app (Figures 3A-3C)
  - Add function to have personal home page or bio area so that users can bookmark information relevant to them (not to be made accessible to anyone else)
  - Add functionality to link users directly with health care professionals to aid communication
  - Add function to include standard needs assessment questionnaires and share this information directly with health care professional teams (refer to Figure 3B for evolved conceptual premise of daily tracker and needs assessment)
  - Add functionality to include information and frequently asked questions relating to local cancer hospital to reduce anxiety as a new patient

## Discussion

### Principal Findings

This project drew upon user-centered and co-design methods to understand the experiences of YAs diagnosed with cancer. Focusing on experiences surrounding diagnosis has provided an understanding of the potential role of a digital intervention to support YAs from the point of cancer diagnosis to treatment commencement—and beyond—with a focus on psychological support and well-being. Prioritizing user-centered input to inform the development of experientially driven features and functionalities has facilitated the development of both a first- and second-generation medium- to high-fidelity prototype of an app aligned to previously identified prehabilitation benefits such as personal empowerment, physical and psychological resilience, and positive impacts on long-term health [16].

The aims and outcomes of this study are in line with local, national, and international digital health and care strategies [17] focused on empowering patients and citizens to engage and manage their own health and well-being. In the United Kingdom, in 2020, MacMillan Cancer Support established an 11-point action plan to ensure the adoption and further development of prehabilitation in cancer care [16]. This action plan focuses on points such as integrating prehabilitation into established clinical pathways; developing local and regional resources for users; developing standardized and validated measures for screening, assessment, and outcomes; and pursuing the research and business agendas.

Successful integration of digital health technologies into care provision pathways and services relies on the adoption readiness of the target end users. Previous work has illustrated that digital health technologies that are not reflective of existing health care pathways or the needs of patients and HCPs can be a preventative barrier to their routine adoption [28]. In this study, we engaged with both patients and HCPs to understand what the barriers to adoption of a new digitally driven supportive care service may be and where in the implementation pathway they may exist.

The importance of involving citizens in the design and development of new technologies and tools to ensure successful integration of digital health into care is a central tenet of digital health and care strategies [17,29]. We know that services are better adopted when co-design strategies have been embedded throughout their development cycle [23]. Involving both patients and HCPs enabled deeper understanding of the varying motivators and barriers to implementing digital solutions in daily practice. Similarly, the iterative approach allowed us to understand the needs of the YAs and HCPs and refine the design and functionality of the app accordingly [23].

This approach is consistent with that of other app development projects in similar population groups [23]. Casillas et al [23], for example, described the development and feasibility testing of an SMS text messaging system to increase adherence to, and receipt of, survivorship care in YA populations. We used a multistage co-design process involving interviews with YAs. Our system was found to be acceptable and feasible to YAs, and it was concluded that it had the potential to improve receipt of survivorship care in this population.

The findings from this project concur with those of studies of a similar nature; for example, Lea et al [30] also conducted participatory research with YAs diagnosed with cancer about their support needs and use of web-based information. The authors found that YAs use a range of social media, medical websites, search engines, charity websites, and communication platforms (eg, WhatsApp) to access information and support. No one source seemed to provide YAs with all the information they need and the ability to connect with peers with similar experiences for additional support. Our work and our Cancer Helpmate prototype app are already going some way to address some of these accessibility issues because we have applied the experiences and feedback directly of the YAs in our study to inform the co-design development of the prototype app.

Elsbernd et al [31] developed an app to support YAs who have received treatment for cancer, using a cocreation process that involved 3 creative group workshops with YAs, HCPs, and researchers. Three key features for the app were identified through this process: (1) a community forum, (2) an information library, and (3) a symptom and side effect tracker. Similar to our project, bright, warm colors were chosen by the YAs as a key design feature. Having the functionality to personalize the content presented to the user was highlighted by participants in this study, which is consistent with the findings of a qualitative study conducted by Linder et al [32], who used a computerized symptom capture tool to understand the symptoms and side effects that YAs with cancer undergoing chemotherapy experience and the self-management methods they use. The authors found that YAs often had similar symptoms and side effects but self-managed them in unique ways.

Lea et al [30] argue the case for developing effective resources collaboratively with YAs to ensure that they support the holistic needs of YAs with cancer. This is consistent with the findings from Siembida et al [33], who, after conducting a survey study among adolescents with cancer on their perceived quality of care, found that patient engagement had no impact on perceived quality of care. Instead, those adolescents who felt that providers supported their independence had a higher perceived quality of care than those who did not. This suggests that it is important to provide YAs the opportunity to give their opinion on, as well as ask questions of, and have input into, their treatment plans. Our Cancer Helpmate app is on a positive trajectory to be able to facilitate this because it contains engaging features and functionalities relevant to holistic and prehabilitative care for YAs with cancer.

### Strengths and Limitations

The key strength of this project is the co-design approach with multiple stages of data collection, which prioritized the views and input of YAs, HCPs, and DHPs. Recruitment was challenging at times, but the research team persevered and identified as many different ways as possible to reach and recruit participants. A responsive approach such as this one did require submission of minor and major ethics protocol amendments during the project to reflect necessary changes to the inclusion criteria and recruitment methods, and these affected the initially conceived project timelines. It may be a limitation that more of the sample of YAs were asked to reflect on their cancer diagnosis experiences up to 3 years after diagnosis, but such is the impact of the diagnosis experience for this population that they were able to articulate and describe this with clarity and detail.

We also actively responded to recruitment challenges by delivering presentations to the clinical team, placing an advertisement in a national professional body newsletter targeting professionals working specifically with the target population, and establishing researcher presence in the YA hospital clinics to speak directly to YAs after the initial identification by, and introduction from, the clinician. This last strategy proved particularly effective because 5 (71%) of the 7 YAs recruited for a user-requirement interview were from the direct meeting with the researcher at the YA clinic. Such was the value of this recruitment strategy that it is advocated as a mechanism for other researchers working with YAs to embed within their own recruitment strategies in the future.

A notable strength of this project is the delivery of a second-generation medium- to high-fidelity prototype app that reflects the needs and requirements of the end users gathered through the multiple data collection streams in the project. However, it is acknowledged that the small sample size in relation to YAs and professionals recruited to the study could be considered a limitation in terms of representativeness of experiences and input. The reasons for these recruitment challenges in this study are not fully understood, but our responsive actions to the recruitment challenges enabled us to engage directly with our target populations. The number and range of professional roles of the clinicians who did participate are, however, somewhat representative of the size of, and multidisciplinary care provided by, the YA cancer team at the partner clinical site, although it is disappointing that there was no nurse representation in our final sample. In addition, funding requirements placed an initial geographic limitation on recruitment and consequently eligible YA participants before ethics protocol amendments allowed us to broaden recruitment scope.

### Conclusions

The cancer diagnosis pathway for some YAs can be a protracted, frustrating, and anxiety-inducing experience. Upon diagnosis, pathways of care can be rapidly activated, and a YA’s health status can change within hours or days. In such cases, YAs receive a substantial amount of new and important information at accelerated pace. We identified through our engagement with YAs in this study that although a range of age-appropriate, age-targeted, good-quality, and, when read, helpful information was provided to YAs from the hospital, this was predominantly delivered via traditional printed materials. Our qualitative interview and survey findings illustrated that this medium and the timing of delivery were often overwhelming for YAs, affecting negatively their engagement with the materials and information.

However, early consensus activities in this study were encouragingly positive about the need for this app; therefore, Cancer Helpmate has scope to augment information and psychosocial support services provided by YA cancer teams in the future and add to their digital service provisions.

To do this, future evaluation and implementation activities of Cancer Helpmate would be informed by, and learn from, the evolving digital health provision for similar populations. In the United Kingdom, for example, an app called Integrated Assessment Mapping has been implemented in some YA cancer services with support from the national Teenage Cancer Trust. The app allows YAs diagnosed with cancer to self-identify their needs through use of a holistic needs assessment to enable their clinical team to identify support needs based on information collected by the system [34]. Indeed, in this study too we recognize the holistic nature of supportive care but in the context of prehabilitation (including diet, exercise, self-care, and well-being) and have developed a prototype app that centralizes, and can personalize, this information for the user. Our next chapter in this program is to engage in making further developmental iterations to the product and move toward testing and evaluation with end users in community and hospital settings. In particular, we are interested in enhancing more of the app personalization components that would be selected by YAs during the onboarding process and evaluate their acceptability and utility. We also anticipate more formally evaluating the impact of written and digital information provision with this population.

## Abbreviations

DHP: digital health professional
HCP: health care professional
YA: young adult

## Data Availability

The data sets generated and analyzed during this study are available from the corresponding author on reasonable request.
